# Supplementary material for: MicroRNA and Protein Biomarkers of Intestinal Permeability in the Assessment of Metabolic Dysfunction-Associated Steatotic Liver Disease (MASLD)
Source: Int J Mol Sci. 2025 Nov 24;26(23):11351. doi: 10.3390/ijms262311351 (PMC12692580; doi:10.3390/ijms262311351)
Supplement: Supplementary file 1 [file ijms-26-11351-s001.zip › ijms-3983491-supplementary.pdf]

Supplementary Materials:

Table S1. Independent indicators of the presence of liver steatosis. Stepwise logistic regression model with backward selection

```
. logistic Group foldmiR122 foldmiR21 GGTP ferrytyna

Logistic regression                                     Number of obs =    153
                                                        LR chi2(4)    =  79.05
                                                        Prob > chi2   = 0.0000
Log likelihood = -57.153037                             Pseudo R2     = 0.4088
```

Group	Odds ratio	Std. err.	z	P>|z|	[95% conf.	interval]
foldmiR122	1.101178	.0484962	2.19	0.029	1.010114	1.200452
foldmiR21	.4939185	.1437633	-2.42	0.015	.2791893	.8737997
GGTP	1.056869	.0173889	3.36	0.001	1.023331	1.091507
ferrytyna	1.008518	.0030313	2.82	0.005	1.002594	1.014476
_cons	.1638083	.1028058	-2.88	0.004	.0478766	.560465

Note: _cons estimates baseline odds.
Note: 0 failures and 1 success completely determined.

Table S2. Independent predictors of advanced steatosis. Stepwise multivariable logistic regression model with backward selection.

```
. logistic AdvancedsteatosisS120S3 ALT AST DAOngml

Logistic regression                                     Number of obs =    101
                                                        LR chi2(3)    =  16.94
                                                        Prob > chi2   = 0.0007
Log likelihood = -60.700715                             Pseudo R2     = 0.1224
```

AdvancedsteatosisS120S3	Odds ratio	Std. err.	z	P>|z|	[95% conf.	interval]
ALT	.9533795	.0215587	-2.11	0.035	.912048	.9965842
AST	1.116083	.0460094	2.66	0.008	1.029453	1.210003
DAOngml	1.026818	.0088665	3.06	0.002	1.009587	1.044344
_cons	.0708964	.0593048	-3.16	0.002	.0137593	.3653031

Note: _cons estimates baseline odds.

Table S3. Independent predictors of significant fibrosis (TE stage F2-F4). Stepwise multivariable logistic regression model with backward selection.

```
. logistic SignificantfibrosisF010F24 ALT AST DAOngml foldmiR122

Logistic regression                                     Number of obs =    100
                                                        LR chi2(4)    =  15.57
                                                        Prob > chi2   = 0.0037
Log likelihood = -34.486396                             Pseudo R2     = 0.1842

------------------------------------------------------------------------------------
SignificantfibrosisF010F24 | Odds ratio   Std. err.      z    P>|z|     [95% conf. interval]
---------------------------+--------------------------------------------------------
                       ALT |   .9504024   .0241234   -2.00   0.045     .9042783    .9988793
                       AST |   1.133933   .0526268    2.71   0.007     1.035339    1.241917
                   DAOngml |   .9871557   .0135012   -0.95   0.345     .9610453    1.013975
                foldmiR122 |   1.000144   .0037592    0.04   0.970     .9928029    1.007539
                     _cons |   .0309987   .0345021   -3.12   0.002      .003499    .2746283
------------------------------------------------------------------------------------
Note: _cons estimates baseline odds.
```

Table S4. Associations between analysed parameters of intestinal permeability.

Variable	**DAO**		**TNFa**		**IL-6**		**LBP**		**miR-122**		**miR-21**		**miR-29a**	
	Rho	P value	Rho	P value	Rho	P value	Rho	P value	Rho	P value	Rho	P value	Rho	P value
LBP	**0.23**	**0.02**	-0.04	0.72	-0.07	0.51	-	-	0.08	0.42	0.10	0.34	0.06	0.52
TNFa	0.07	0.49	-	-	-0.01	0.90	-0.04	0.72	**0.36**	**0.0002**	**0.27**	**0.006**	0.12	0.21
IL-6	0.04	0.67	-0.01	0.90	-	-	-0.06	0.51	-0.14	0.17	-0.08	0.44	-0.06	0.55
DAO	-	-	0.07	0.49	0.04	0.67	**0.23**	**0.02**	-0.07	0.49	-0.05	0.65	-0.05	0.62
foldmiR21	-0.05	0.65	0.27	0.006	-0.08	0.44	0.10	0.34	0.73	<0.0001	-	-	**0.77**	**<0.0001**
foldmiR122	-0.07	0.49	0.36	0.0002	-0.14	0.17	0.08	0.42	-	-	**0.73**	**<0.001**	**0.48**	**<0.0001**
foldmiR29a	-0.05	0.62	0.12	0.21	-0.06	0.55	0.06	0.52	0.48	<0.0001	**0.77**	**<0.001**	-	-
